# Oxidant Sensing by Protein Kinases A and G Enables Integration of Cell Redox State with Phosphoregulation

**DOI:** 10.3390/s100402731

**Published:** 2010-03-26

**Authors:** Joseph R. Burgoyne, Philip Eaton

**Affiliations:** Department of Cardiology, The Rayne Institute, King’s College London, St. Thomas’ Hospital, London, SE1 7EH, UK; E-Mail: joseph.burgoyne@kcl.ac.uk

**Keywords:** protein kinase A, protein kinase G, oxidation

## Abstract

The control of vascular smooth muscle contractility enables regulation of blood pressure, which is paramount in physiological adaptation to environmental challenges. Maintenance of stable blood pressure is crucial for health as deregulation (caused by high or low blood pressure) leads to disease progression. Vasotone is principally controlled by the cyclic nucleotide dependent protein kinases A and G, which regulate intracellular calcium and contractile protein calcium sensitivity. The classical pathways for activation of these two kinases are well established and involve the formation and activation by specific cyclic nucleotide second messengers. Recently we reported that both PKA and PKG can be regulated independently of their respective cyclic nucleotides via a mechanism whereby the kinases sense cellular oxidant production using redox active thiols. This novel redox regulation of these kinases is potentially of physiological importance, and may synergise with the classical regulatory mechanisms.

## 1. Introduction

The importance of blood pressure is eminently clear in the modern western world where poor diet and lifestyle has lead to a dramatic rise in individuals with high blood pressure (hypertension). Untreated, this can lead to increased risk of pathological complications including heart attacks, heart failure, peripheral artery disease, aortic aneurysms, stroke and kidney failure [1]. To prevent these complications, hypertension should be rapidly diagnosed and treated. Low blood pressure (hypotension) can also be detrimental to health, especially when it results in inadequate tissue perfusion and end organ damage. This is often the case in patients with sepsis where hypotension is a symptom of this disease resulting from bacterial infection [2,3]. Due to the harm of sustained hypo- or hypertension, humans have evolved numerous biochemical pathways for regulating blood pressure, permitting dynamic changes in blood flow to occur, thus allowing the body to adapt to physical and environmental changes. However, these pathways can become disrupted as a result of genetic susceptibility and lifestyle factors, leading to loss of blood pressure homeostasis and disease progression. Over many years the underlying mechanisms that regulate blood pressure have been elucidated, giving us a greater understanding of the biological processes that can lead to dysfunction. This increase in knowledge has lead to the development of numerous drugs that can help prevent hypertension including angiotensin-converting enzyme (ACE) inhibitors, beta blockers, diuretics, calcium channel blockers and angiotensin II receptor antagonists [1]. Although much is known about blood pressure regulation, the field is still advancing with the potential for more effective drug targets and treatments. Indeed, the discovery that protein kinase A (PKA) and protein kinase G (PKG) are oxidant sensors that can regulate blood pressure and cardiac contractility via a novel redox mechanism offers the potential for novel drugs that activate these pathways [4,5]. In this article the biochemical mechanisms by which PKA and PKG regulate blood pressure and cardiac contractility are discussed, with a detailed consideration of the newly discovered redox mechanism by which they can be enzymatically regulated.

**Figure 1.**
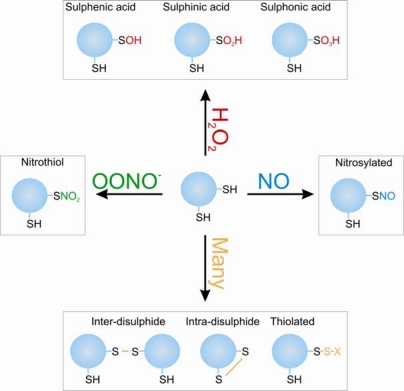
The oxidative post-translational modifications that can form on cysteine residues.

Oxidants are emerging as important physiological signalling molecules despite many years of bad press, which erroneously labeled them as being purely causative agents in disease progression. This turn around in perception is due to the failure of numerous antioxidant trials and the growing discovery of proteins and pathways that are oxidatively regulated (the “redoxome”) [6–8]. A great variety of redox sensitive proteins have been identified including kinases, phosphatases, transcription factors, ion channels, metabolic enzymes, RNA binding proteins, caspases and N-acetyl transferases [9]. Protein kinase A and G belong to this sub-population of proteins that can act as oxidative sensors due to their ability to be modified and enzymatically regulated by cellular oxidants. These proteins contain “reactive” cysteine thiols, which are those stabilised in the deprotonated more reactive thiolate (RS^−^) form due to their local environment. Close proximity with the basic amino acids arginine or lysine lower the pKa of cysteine thiols making them more reactive. These thiols can act as redox sensors by undergoing a multitude of different oxidative modifications as summarised in Figure 1, which is dependent on the oxidant present and proximity to other reactive thiol containing proteins or molecules. This process of protein oxidation can regulate cell signalling by altering protein function due to a structural change generated by the distinct shape and charge characteristics of the oxidative modification. Many forms of these post-translational oxidative modifications can be readily reversed by cellular reducing enzymes such as thioredoxin, peroxiredoxin and glutaredoxin [10], allowing dynamic reversible signalling events to occur analogous to that of phosphorylation.

The generation of the reactive oxygen species superoxide is a continual process due to its formation as a by-product of energy metabolism crucial for cellular survival and homeostasis [11,12]. Furthermore, there are several non-metabolic enzymes that also generate superoxide as a by-product of catalysis, many of which are oxidases that reduce molecular oxygen. A group of enzymes also exist that generate superoxide as a primary product, and are known as NADPH oxidases. These enzymes are known to play an important role in both physiological as well as pathological signalling in cell growth, migration and tissue inflammation [13]. The superoxide generated by these enzymes can be readily converted to the more stable oxidant hydrogen peroxide (H_2_O_2_) following reduction by superoxide dismutase [14]. This form of ROS acts as a physiological second messenger signalling molecule by selectively oxidising target proteins [9]. H_2_O_2_ is able to act as a signalling molecule by fulfilling the several criteria required for the dynamic regulation of protein function. This includes regulated formation of this oxidant in response to specific stimuli, selectivity of target protein oxidation (provided by “reactive” cysteine residues only in selected targets), a change in protein function (that must be demonstrated experimentally), and rapid reversibility by reducing enzymes, as well as localised changes in cellular redox due to compartmentalisation of antioxidants and oxidant-generating enzymes. This has been shown to be the case for oxidants generated transiently in response to growth factor and cytokine stimulation, where their formation is essential for the full spectrum of signalling events they can trigger [15–17].

## 2. Regulation of Vascular Smooth Muscle Tone

The regulation of blood pressure is maintained physiologically by changes in smooth muscle tone. Increased relaxation causes vessels to dilate, augmenting the total volume blood capacity, leading to a subsequent decrease in pressure within the blood vessel. Substantial characterisation at the molecular level has revealed that vascular smooth muscle tone is essentially controlled by the interplay between two opposing pathways, with the overall result being dependent on the rate of myosin light chain phosphorylation. The contractile response is calcium dependent and leads to increased myosin light chain kinase (MLCK) activity and inhibition of the myosin light chain phosphatase (MLCP) [18]. This increases the phosphorylation state of the myosin light chain thereby stimulating its ATPase activity, resulting in augmented contraction due to increased cycling of cross-bridges with actin. Several circulating factors are known to induce smooth muscle contraction including endothelin-1 [19,20], angiotensin II [21], and the alpha1-beta adrenergic receptor stimulators epinephrine and norepinephrine [22,23]. Activation of specific receptors on the surface of smooth muscle cells by these ligands leads to increased G protein dependent activation of phospholipase C [24]. This enzyme cleaves phosphatidylinositol 4,5 bisphosphate (PIP2) to form D-myo-inositol-1,4,5-trisphosphate (IP_3_), a second messenger that increases Ca^2+^ release from the sarcoplasmic reticulum (SR) by activating IP_3_ receptors (IP3R). This enhances Ca^2+^ dependent Ca^2+^ entry via opening of transient receptor potential channels (TRPs) and SR store-operated Ca^2+^ channels [25,26]. This elevated cytosolic Ca^2+^ interacts with calmodulin forming a complex that activates MLCK, a process which leads to enhanced vascular smooth muscle constriction [27].

**Figure 2.**
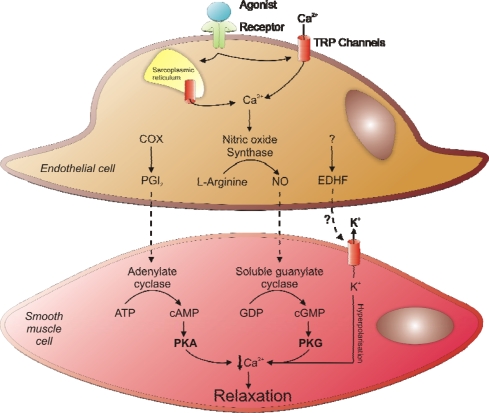
Three dominant pathways for regulating vascular smooth muscle relaxation.

The contractile response of vascular smooth muscle is inhibited by three known converging pathways that prevent myofilament cross-bridge cycling to generate smooth muscle relaxation. These are the Protein kinase A (PKA), Protein kinase G (PKG) and endothelium derived hyperpolarising factor (EDHF) pathways, each of which acts as a vasodilator by decreasing intracellular Ca^2+^ in vascular smooth muscle cells. This decrease in Ca^2+^ concentration leads to inhibition of MLCK and activation of MLCP, switching the myosin light chain from the phosphorylated contractile state to the dephosphorylated relaxed state. The three known vasodilatory pathways are summarised in Figure 2.

Both PKA and PKG are classically considered to be cyclic nucleotide dependent and require the formation of active vasodilators by the endothelium. In the case of PKA, increased prostacyclin (PGI_2_) formation and diffusion from endothelial cells to smooth muscle cells increases the activity of adenylate cyclase, which converts adenosine triphosphate (ATP) to 3′–5′-cyclic adenosine monophosphate (cAMP) a direct activator of PKA [28]. For the classical activation of PKG, nitric oxide (NO) is generated by nitric oxide synthases (NOS) located in endothelial cells. The signalling molecule NO then diffuses into smooth muscle cells where it binds to the haem centre and activates soluble guanylate cyclase, an enzyme that converts guanosine triphosphate (GTP) to 3′,5′-cyclic guanosine monophosphate (cGMP), a secondary messenger that directly activates PKG [29].

Formation of the vasodilator NO in endothelial cells is mediated by shear stress as well as a number of biological circulating factors including bradykinin, substance P, acetylcholine, histamine, adenosine and thrombin [30,31]. These substances all generate smooth muscle relaxation by binding to G-protein coupled receptors on the surface of endothelial cells, which line the inner wall of blood vessels. Activation of these receptors induces the production of IP_3_ by phospholipase C. This secondary messenger binds to IP3R on the surface of the endoplasmic reticulum, causing the release of Ca^2+^, which then binds to calmodulin, forming a complex that stimulates the activity of NOS [30]. This enzyme utilises arginine to produce NO and the by-product citrulline.

Activation of PKG in smooth muscle cells leads to phosphorylation of several targets that regulate contractile protein Ca^2+^ sensitivity and the intracellular Ca^2+^ concentration. This includes direct modification of the large conductance Ca^2+^-activated K^+^ channel (BKca) on the cell membrane, increasing its open probability [32,33]. Increased cellular export of K^+^ cation causes membrane hyperpolarisation, closing voltage-dependent Ca^2+^ channels, decreasing the total Ca^2+^ influx. Another source of intracellular Ca^2+^ is that extruded from the SR with the influx and efflux being regulated by several specific membrane Ca^2+^ handling proteins. This includes the Ca^2+^ importing pump SERCA, which is normally inhibited by the associated protein phospholamban. Phosphorylation of phospholamban by PKG removes the brake on SERCA activity, increasing the import of Ca^2+^ into the SR [34,35].

Most contractile agonists work by activating smooth muscle G_q_-coupled receptors and phospholipase C to increase IP_3_ production. This second messenger stimulates Ca^2+^ release from the SR, which activates MLCK. PKG is able to inhibit this pathway by phosphorylating the IP_3_ receptor-associated PKG-substrate (IRAG), which when modified inhibits IP_3_ stimulated SR Ca^2+^ release by the IP3R [36–38]. There is also some evidence that PKG can inhibit the IP3R by direct phosphorylation [39]. Furthermore, the production of IP_3_ by phospholipase C is suppressed by PKG-dependent phosphorylation of the regulator of G-protein signalling 2 (RGS2) [40,41]. When RGS2 is phosphorylated by PKG it increases the GTPase activity on G_q_, decreasing the GTP that is required for phospholipase C activation.

The activation of MLCP is crucial for smooth muscle relaxation, which is maintained by PKG-dependent target phosphorylation. Essentially PKG is able to do this by preventing the inactivation of MLCP by Rho kinase (ROCK). The activator of ROCK, rhoA is directly phopshorylated by PKG increasing its affinity for the guanine dissociation inhibitor (GDI), preventing the translocation of RhoA to the membrane which is required for activation [42,43]. Another target of phosphorylation by PKG is Ser-695 on the myosin phosphatase target subunit 1 (MYPT1) of the MLCP, which prevents ROCK from phosphorylating the adjacent Thr-696 that normally leads to enzyme inactivation [44,45]. An additional target of PKG is the small heat shock protein HSP20 which induces smooth muscle relaxation and blocks agonist induced constriction [46,47]. HSP20 acts as a late-phase signalling molecule, binding to actin when phosphorylated by PKG, which likely prevents reorganisation of the actin cytoskeleton that is required for constriction.

The enzymes PKA and PKG share much homology in terms of structure especially around the catalytic site, giving both kinases the ability to phosphorylate several common substrates. Variations in targets between the two kinases is likely due to differential localisation controlled by binding proteins, such as A-kinase anchoring proteins (AKAPs) for PKA and G-kinase anchoring proteins for PKG (GKAPs) [48–51]. These anchoring proteins localise the kinases to the vicinity of their substrates, allowing controlled selective signalling events to occur. Like PKG, PKA activation also induces smooth muscle relaxation [52]. However the mechanism for PKA has not been characterised to the same extent to that of PKG. Nevertheless several shared substrates have been identified between the two kinases which mediate vasodilation. These include phospholamban [53,54], HSP20 and RhoA [46,55,56], with some evidence of MYPT1 also being a target of PKA [57].

A third biochemical process that mediates vascular dilation is the formation of EDHF, which as the name suggests is produced in endothelial cells and generates relaxation in smooth muscle cells by hyperpolarising the membrane. The synthesis of EDHF is stimulated by the neurotransmitter acetylcholine, the small peptide bradykinin, shear stress and a number of other agonists. The role of EDHF in regulating blood pressure is as prominent as the PKG and PKA pathways, with deficiency in EDHF signalling contributing to several cardiovascular pathologies including hypertension, chronic renal failure, and diabetes [58,59]. The reason for this is likely due to EDHF being particularly effective in modulating vascular tone in an endothelium-dependent manner in small resistance vessels, which are the major determinants of systemic blood pressure [60–62]. The mechanism by which EDHF induces vasodilation is independent of NO and PGI_2_ with its exact identity remaining unknown [58]. However, several potential candidates for EDHF have been proposed including K^+^, epoxyeicosatrienoic acid (EET), H_2_O_2_ and electronical communication through the gap junctions [59,63–65]. Evidence exists for each being an EDHF but its identity (although it likely has multiple components) remains controversial due to substantial contradictory evidence. The failure to get consistent results between different research groups is likely due to variations in the experimental conditions or the use of different vessel types that may have differential responses to each proposed EDHF.

The classical view of blood pressure regulation involves a summation of three primary pathways that are coordinated in a complex manner as outlined above. These pathways are independently regulated but also integrate to generate a net blood pressure. Each pathway can be dynamically regulated by changes in circulating factors and in some cases shear stress with responses varying depending on the specifics of the diverse physiological/pathological conditions and between different vessel types. The inhibition of EDHF signalling or knockdown of PKG causes dysfunctional regulation of blood pressure, which highlights the importance of each pathway in controlling vasotone [58,59,66]. The significance of PKA signalling in regulating blood pressure has not been characterised to the same extent and therefore its overall contribution is not so apparent. Our discovery of a novel mode for the oxidative regulation of the PKA and PKG pathway (as summarised in section 4) further adds to the complexity and potential dynamic regulation of blood pressure. This discovery highlights the potential for cross-talk between these pathways and possible integration of the EDHF phenomenon into the PKG signalling cascade.

## 3. Regulation of Cardiac Contractility

As with the control of vascular smooth muscle cell contraction and relaxation, regulation of cardiac contractility is also dependent on intracellular calcium. Increased Ca^2+^ influx into cardiac cells stimulates the myofilaments to generate more force during contraction due to greater cross-bridge cycling. This process is carefully maintained to ensure adequate myocardial contractile performance and is primarily regulated by the autonomic nervous system, allowing the heart to adapt and function effectively during various environmental challenges [67]. This includes regular daily activities such as aerobic exercise and even during simple tasks such as the body assuming an upright position. These are often termed the “flight or fight” response and are characteristic of a change in heart rate, force of cardiac contractility and the rate of subsequent cardiac relaxation. Stimulation of the sympathetic nervous system leads to release of β-adrenergic receptor agonists from activated sympathetic nerves into the circulatory system [68]. The stimulation of β-adrenergic receptors in the heart by these agonists activates adenylate cyclase (AC) via the stimulatory G-protein (G_Sα_), this increases cAMP production and subsequent PKA activation [68,69].

The heart is a large pump that generates the force required for propelling the blood through the circulatory system by contracting and relaxing in a rhythmic fashion (sinus rhythm). The initiation of cardiac muscle contraction is controlled by the sinoatrial node (cardiac pacemaker) [70], which is located in the upper wall of the right atrium and initiates atrial contraction by generating a wave of electrical excitation (action potential). This electrical stimulation moves through the atria until it reaches the atrioventricular node, situated in specialised tissue between the atria and the ventricles of the heart (posteroinferior region of the interatrial septum). The impulse is delayed at this node to allow enough time for the atria to eject its blood into the ventricles first, before the ventricles contract. After the delay the wave of stimulation is transmitted through the bundles of His and back up the Purkinje fibres causing contraction of the ventricles. The process from electrical excitation of the myocyte to contraction of the heart is known as cardiac excitation–contraction coupling. Each heart muscle cell contracts in unison when stimulated by the action potential [71]. In the unstimulated (resting potential) state the muscle cell sarcolemma (plasma membrane) is polarised. This is due to large negatively charged intracellular proteins and amino acids and the balance between K^+^ inside and Na^+^ outside the cell. This gives the sarcolemma a negative charge on the inside with respect to the outside. During an action potential opening and then rapid closure of Na^+^ channels on the sarcolemma leads to depolarisation and activation of voltage-dependent Ca^2+^ channels, including the L-type Ca^2+^ channel located in the T-tubular membrane. This increases the influx of Ca^2+^ and the inward Ca^2+^ current (*I*Ca), which contributes to the action potential plateau. The elevated Ca^2+^ binds to and activates the ryanodine receptors (RyRs), which are ligand-gated channels located on the SR membrane. This enhances the release of Ca^2+^ from the SR and is known as Ca^2+^-induced Ca^2+^ release. This amplified intracellular Ca^2+^ binds to cardiac troponin C (cTnC) triggering the constriction of myofilament proteins. The removal of Ca^2+^ from the cytosol allows dissociation of Ca^2+^ from troponin C and subsequent myofilament relaxation [72]. This occurs during repolarisation of the sarcolemma due to opening of K^+^ channels, increasing the export of K^+^, closing the voltage-dependent Ca^2+^ channels. The intra and extracellular Na^+^ and K^+^ concentrations are returned to resting state by the sarcolemmal Na^+^/K^+^-ATPase (NKA). The extrusion of Ca^2+^ from the cytosol is mediated by the SR Ca^2+^-ATPase (SERCA), the sarcolemmal Na^+^/Ca^2+^ exchange (NCX1) and sarcolemmal Ca^2+^-ATPase. Increased cytosolic Ca^2+^ is sequestered into the SR Ca^2+^ store by SERCA, replacing that lost from the RyR during an action potential [73]. Extrusion of Ca^2+^ from the cell is mediated by NCX1, which is generally believed to import three Na^+^ ions for every Ca^2+^ ion extruded and is regulated by both transmembrane voltage and Na^+^ and Ca^2+^ concentration gradients [74]. This exchanger can work both in forward and reverse mode with evidence suggesting during the plateau phase of an action potential it extrudes Na^+^ and imports Ca^2+^, with it flipping to the forward mode during repolarisation [75–77]. A second mechanism that has a minor role in the export of Ca^2+^ from the cell is the sarcolemmal Ca^2+^-ATPase, which hydrolyses ATP to transport Ca^2+^ out of the cell [78].

**Figure 4.**
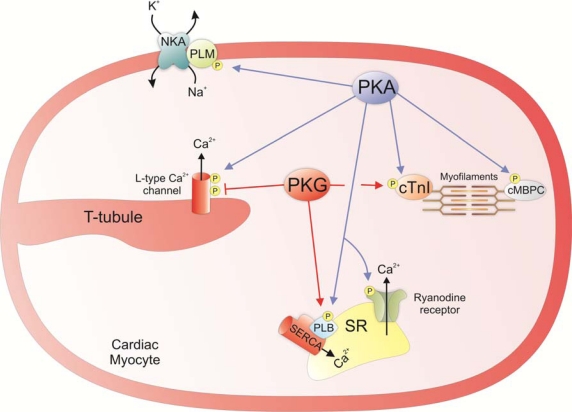
The biochemical targets of PKA and PKG in cardiac myocytes that regulate cardiac contractility.

Phospho-regulation by PKA of several key Ca^2+^ handling proteins increases cardiac contractility (see Figure 4). This includes the increased import and export of Ca^2+^ from the SR. One of the targets of PKA located on the SR is the Ca^2+^ channel the ryanodine receptor. This protein is a tetramer comprised of four type 2 RyR polypeptides (RyR2) and four FK506 binding proteins (FKBP12.6). The activity of this protein is regulated by intracellular Ca^2+^ with low concentrations (μM) increasing activity and high concentrations (mM) being inhibitory [79]. PKA is able to regulate the open probability of RyR by phosphorylating RyR2 polypeptides causing them to dissociate from FKBP12.6. This increases the open probability and enhances sensitivity to Ca^2+^-induced activation. With the overall effect being the augmented export of Ca^2+^ from the SR following an action potential [80]. Another SR Ca^2+^ handling protein that is regulated by PKA is the SERCA pump. The activity of this protein is inhibited by its interaction with the 52 amino acid membrane protein phospholamban. Phosphorylation of phospholamban at Ser-16 by PKA attenuates its ability to inhibit the SERCA pump [81]. This increases the import of Ca^2+^ into the SR, which enhances the rate of relaxation and also results in augmented release of Ca^2+^ following a second action potential from the enhanced Ca^2+^ SR store. The influx of extracellular Ca^2+^ is also enhanced by PKA through the regulation of sarcolemmal Ca^2+^ handling proteins. This includes the L-type Ca^2+^ channel that is phosphorylated by PKA at the α_1C_ subunit at Ser1928 and the β_2_ subunit at Ser-478 and Ser-479, which increases the open probability of this channel [82,83]. The activity of the Na/K ATPase is also regulated by PKA. Phosphorylation of the associated protein phospholemman (PLM) at Ser-68 by PKA relieves its ability to inhibit the NKA, increasing the cellular export of Na^+^ [84].This is negatively inotropic which is contradictory to the effect that is normally associated with PKA activation. However it is believed that increased activity of the NKA by PKA is important in preventing Ca^2+^ overload in response to β-stimulation by limiting the rise in intracellular Na^+^ that may help maintain the NCX1 in its forward mode. This is supported by transgenic mice that do not express PLM in which β-adrenergic stimulation does not activate NKA and that the amplitude of Ca^2+^ transients is significantly higher than for wild-types. The SR Ca^2+^ content was also increased in PLM knockout mice and was associated with an increased propensity for spontaneous Ca^2+^ transients and contractions in these animals [85]. Furthermore it has been suggested that NKA activation by PKA may also prevent diastolic dysfunction and protect against arrhythmias although further research is required to substantiate these claims.

As well as Ca^2+^ handling proteins, PKA also has several targets that are myofilament proteins required for cardiac constriction. Cardiac troponin I (cTnI) is a target with residues 23 and 24 being phosphorylated. This increases the rate of cardiac relaxation by enhancing the rate at which Ca^2+^ is dissociated from cTnC without effecting maximum actomysoin ATPase activity [86–88]. Another myofilament target of PKA is the cardiac myosin binding protein C (cMyBP-C). Using transgenic mice that have a non-phosphorylatable form of cMyBP-C it was found that basal phosphorylation is necessary for maintaining thick-filament orientation, dynamic regulation, and contractile mechanics [89]. This basal phosphorylation of cMyBP-C is likely maintained by PKA activity preventing cardiac dysfunction. In addition, phosphorylation of this myofilament protein has recently been shown to play a role in enhancing cardiac contractility by accelerating crossbridge kinetics [90].

Activation of PKG is also known to regulate cardiac contractility. However, its role has not been characterised to the same extent as PKA and is seen as somewhat controversial due to the biphasic effect of NO donors on the cardiac inotropy. Nevertheless a recent publication has put into perspective the likely effect of direct PKG stimulation on cardiac contractility. It is reported that NO donor concentrations that induce protein oxidation (S-nitrosylation) and not PKG activation increase cardiac contractility. In contrast, concentrations of NO donor that stimulate PKG, or the use of cGMP-analogs as direct activators, lead to decreased contractility [91]. These findings are supported by PKGI knockout mice that do not have a change in force of contraction after treatment with a cGMP-analog PKG activator. In contrast the wild-type mouse shows a reduction in force of contraction following PKG activation [92]. The ability of PKG to decrease contractility is likely through its ability to increase SERCA activity which will lower free cytosolic Ca^2+^ [93,94], and the phosphorylation of cTnI that will decrease the sensitivity of cTnC for Ca^2+^ [95,96]. Both of these proteins are substrates for PKA and PKG but the reason for the difference in contractility between these kinases is that unlike PKA, PKG does not also simultaneously enhance the positively inotropic SR release of Ca^2+^ or extracellular influx of Ca^2+^. Furthermore, PKG is able to reduce the cellular intake of Ca^2+^ by directly inhibiting voltage activated Ca^2+^ channels. This is substantiated by experiments demonstrating that activation of PKG causes inhibition of L-type Ca^2+^ channel whole cell current after phosphorylation of the ß_2a_ subunit at Ser496 [97]. This is substantiated by previous findings in which mice overexpressing PKGI had increased inhibition of L-type Ca^2+^ channels compared to wild-type [98].

## 4. Redox Regulation of PKAI and PKG1α Activity

As mentioned in the introduction many proteins can act as redox sensors and signal transducers by being functionally regulated by cysteine oxidation. We undertook a project to identify such proteins with the aim of finding those that specifically formed inter-protein disulphide complexes, an anticipated oxidation product [99]. The rationale being that redox signalling may be mediated by oxidants that can induce inter-protein disulphides, a structural modification that has the propensity to alter enzyme function. Detection of these disulphide bound complexes was achieved using a diagonal gel electrophoresis technique. In brief, samples were prepared from ventricular myocytes exposed to control conditions or oxidative stress with the thiol-selective oxidant diamide. Cells were reconstituted in non-reducing SDS sample buffer and resolved by SDS-PAGE. Once complete an entire protein-containing lane was carefully excised and placed horizontally on top of a fresh gel and resolved under reducing conditions. Any proteins that did not form disulphide complexes run at the same molecular weight as during the first separation, forming a diagonal across the gel after being resolved. Proteins that formed a disulphide now run at a lower molecular weight than in the first run and therefore resolve away from the diagonal plane in each gel. Treatment of hearts with diamide increased the number of proteins running off of the diagonal plane in gels stained with colloidal Coomassie blue, representing proteins that formed disulphide bonds. These protein spots were carefully extracted from each gel and analysed using mass spectrometry analysis. Several proteins were identified including the type I regulatory subunit of protein kinase A (PKA-RI). Due the importance of this protein in the regulation of the cardiovascular system, as highlighted above, we investigated the significance of disulphide bond formation on PKAI enzyme function [5]. This kinase was already thought to contain two constitutive disulphides between each RI subunit [100], which run anti-parallel to each other. Thus, cysteine 17 on one subunit forms a disulphide with cysteine 38 on the other, and this occurs twice in each dimer. We hypothesised that these disulphides occurred under oxidising conditions and were not present under basal, reducing conditions present intracellularly. To assess this, we ran non-reducing SDS-PAGE gels using homogenates from hearts perfused with varying concentrations of H_2_O_2_. When these samples were immunostained following Western blotting with a PKA-RI antibody most of the protein was monomeric in the controls, with a concentration-dependent increase in disulphide dimer after H_2_O_2_ treatment. The disulphide reducing agent 2-mecaptoethanol confirmed that the dimer was the result of disulphide formation as it reduced this complex back to its monomeric form.

To determine the functional significance of this oxidative modification on PKA activity the contractility of isolated adult rat ventricular myocytes was measured. In the presence of H_2_O_2_ myocyte contractility was augmented, which could be inhibited by the addition of the PKA inhibitor, H89. This enhanced contractility was accompanied by an increase in phosphorylation of the PKA substrates phospholamban and troponin I, a process which was also sensitive to H89 inhibition. Furthermore, this apparent activation of PKA by oxidation was independent of cAMP. This was evident from a specific fluorescence based assays for cAMP, showing that H_2_O_2_ treatment did not augment formation of this cyclic nucleotide. Together this data suggested that oxidation can directly enhance the activity of PKAI independent of cyclic nucleotide stimulation. We hypothesised that this augmentation of PKAI substrate phosphorylation following oxidation may be mediated by the kinases ability to interact with its binding partners the AKAPs [48,49]. The rational being that formation of redox sensitive disulphides located in the known region where the kinase interacts with its AKAPs may increase binding affinity, and therefore enhance PKAI localisation to its substrates. This was substantiated by the discovery that oxidation of PKA-RI increased its affinity for the AKAP α-myosin heavy chain. This binding protein likely increases localisation of PKAI to its known myofilament substrates, troponin I and myosin binding protein C. Recently the ability of disulphide bond formation in PKA-RI to enhance its affinity for AKAPs has been further substantiated using fluorescence anisotropy experiments. It was shown that mutation of either of the redox sensitive cysteines (so it cannot form a disulphide) decreased the enzymes ability to bind to D-AKAP2 compared to the wild-type protein [101].

**Figure 5.**
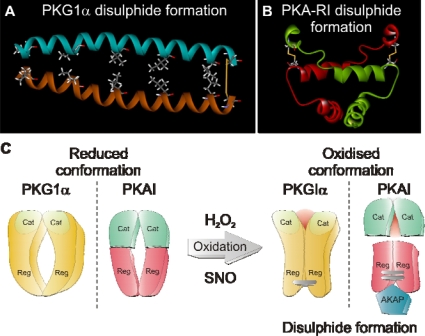
(a) The NMR structure of the N-terminal dimerisation domain of PKG1α including the redox sensitive disulphide bond (b) The NMR structure of the dimerisation domain of the type I alpha regulatory subunit of protein kinase A including the redox sensitive disulphides (c) The basic mechanism of how oxidants (H_2_O_2_ or nitrosylation species of NO (SNO)) can augment PKG1α activity and enhance the affinity of PKAI for it binding partners, the AKAPs.

The discovery that PKAI could be enzymatically regulated by oxidation independent of cyclic nucleotide stimulation lead us to speculate that PKG1α may also be redox sensitive. The rational being that both kinases share similar structural homology and also a single constitutive disulphide within the N-terminal dimerisation domain of PKG1α has been identified [102]. Western blot analysis of hearts perfused using the Langendorff technique with H_2_O_2_ highlighted mostly monomer in controls and a concentration dependent increase in PKG1α dimer formation which mirrored that of PKA-RI [4]. In hearts perfused with H_2_O_2_ there was a substantial time dependent decrease in perfusion pressure analogous to that of an NO donor. We speculated that this decrease in perfusion pressure by H_2_O_2_ may be due to activation of PKG1α by direct oxidation. We tested this hypothesis by measuring tension in aortic rings as readout of vascular tone. The use of the PKG inhibitor Rp-8-Br-cGMPs and the guanylate cyclase inhibitor ODQ blocked NO donor mediated vessel dilation. However, H_2_O_2_ induced vessel relaxation was only blocked by the PKG inhibitor, supporting an oxidative cyclic nucleotide independent mechanisms for PKG1α activation. Michaelis Menten kinetic analysis of recombinant PKG1α activity using a radioactivity based kinase assay confirmed that oxidation can directly activate PKG1α independent of cGMP, by increasing the enzyme affinity for its substrate by approximately 7-fold. Mutation in PKG1α of the redox sensitive cysteine 42 to a charge conserved serine residue removed the ability of this kinase to be activated and to disulphide dimerise in response to H_2_O_2_ treatment. This is compelling evidence demonstrating that oxidation of cysteine 42 is crucial for redox activation of PKG1α. The mechanism for oxidative activation of PKAI and PKG1α is summarised in Figure 5, which also includes the NMR structure for the N-terminus of each kinase with the redox sensitive disulphides highlighted in yellow [103,104].

To some researchers the use of exogenous H_2_O_2_ is sometimes seen as being a non-physiological means of investigating protein oxidation. To overcome the potential skepticism associated with using exogenous oxidants we treated rat aortic smooth muscle cells with physiologically relevant concentrations of insulin, a known mediator of endogenous oxidant formation [105,106]. The use of insulin was sufficient to induce a robust detectible increase in PKG1α disulphide dimerisation, demonstrating that our findings were not an artifact of using exogenous H_2_O_2_. Furthermore it was found that transnitrosylating species of NO (CysNO) are also able to mediate the formation of PKG1α and PKA-RI disulphide dimers [107]. A finding that coincided with increased relaxation in vessels treated with CysNO, which was cyclic nucleotide independent. With the stimulation of PKA by oxidation, one would expect an increase in cardiac contractility that is normally associated with activation of this kinase. However this is not the case when perfusing with H_2_O_2_, which has very little effect on cardiac contractility. As discussed in this article PKA and PKG have opposite effects on the cardiac ionotropy due to their ability to differentially regulate intracellular Ca^2+^. Based on this evidence we hypothesised that simultaneous activation of PKG may override the effect of PKA stimulation. We tested this theory using isoprenaline to activate PKA and enhance cardiac contractility. The addition of an NO donor to stimulate PKG activity reversed the ionotropic effect of isoprenaline, demonstrating that PKG can override PKA induced elevations in contractility. It is important to consider that in perfusion experiments, using an exogenous oxidant will simultaneously activate both kinases due to global cellular oxidation. During a physiological or pathological scenario change in oxidant formation are likely to occur in localised areas of the cell within close proximity to the enzymes that generate them, such as superoxide dismutase. This could allow differential changes in oxidant mediated contractility to occur due to stimulation of select pools of PKA or PKG1α.

The potential physiological importance of oxidative activation of PKG1α or PKAI may tie in with EDHF signalling, with substantial evidence in favor of the oxidant H_2_O_2_ being at least a component of EDHF. This is supported by reports showing that the application of exogenous H_2_O_2_ can lead to relaxation in a number of blood vessels [108–110]. The possibility that H_2_O_2_ acts as a component of EDHF is further supported by NO- and prostanoid-independent relaxation being blocked by catalase, which selectively breaks down H_2_O_2_ [111,112]. In addition, aminotriazole (which inhibits catalase) relieves the inhibitory effect of catalase on EDHF-mediated relaxation [113]. The production of H_2_O_2_ has also been directly measured in porcine coronary microvessels using electron spin resonance imaging following bradykinin treatment (which is known to generate EDHF) [111,112,114]. This intervention induced a detectable increase in the formation of H_2_O_2_ and vessel relaxation, both of which were inhibited by catalase. In theory oxidative activation of PKG1α should mimic the effect of EDHF by increasing the open probability of BK_Ca_ channels after phosphorylation, leading to increased hyperpolarisation and vascular relaxation [32,33]. Therefore the oxidative mechanism for PKG1α or PKAI activation provides a potential molecular explanation for the EDHF phenomenon.

## 5. Conclusion

The discovery that PKAI and PKG1α are redox sensors and signal transducers that can be regulated by cellular oxidants means there needs to be an addition to the well-established models for their activation. These findings highlight the potential for oxidants to regulate both vasotone and the cardiac inotropic environment. However, further characterisation to determine the full extent of the physiological as well pathological roles of these oxidative mechanisms needs to be undertaken. It is tantalising to suggest that EDHF may be an oxidant that can induce vasodilation through direct oxidative activation of PKG1α or PKAI. However, this is yet to be determined and will likely require the development of sensitive techniques that can determine small variations in PKG1α or PKAI oxidation.

## Figures and Tables

**Figure 3.**
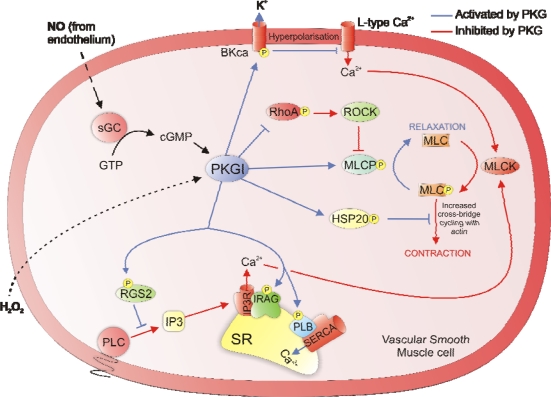
The biochemical targets of PKG in vascular smooth cells that mediate vasorelaxation.
